# Effect of Vitamin D Supplementation on Inflammatory Biomarkers in School-Aged Children with Attention Deficit Hyperactivity Disorder

**DOI:** 10.1155/2022/1256408

**Published:** 2022-08-22

**Authors:** Mahsa Samadi, Fatemeh Gholami, Marzieh Seyedi, Mahmoud Jalali, Mohammad Effatpanah, Mir Saeid Yekaninejad, Mina Abdolahi, Maryam Chamari, Niyaz Mohammadzadeh Honarvar

**Affiliations:** ^1^Department of Cellular and Molecular Nutrition, School of Nutritional Sciences and Dietetics, Tehran University of Medical Sciences, Tehran, Iran; ^2^School of Medicine, Ziaeian Hospital, International Campus, Tehran University of Medical Sciences, Tehran, Iran; ^3^Department of Epidemiology and Biostatistics, School of Public Health, Tehran University of Medical Science, Tehran, Iran; ^4^Department of Community Nutrition, School of Nutritional Sciences and Dietetics, Tehran University of Medical Sciences, Tehran, Iran

## Abstract

**Method:**

This randomized double-blind, placebo-controlled trial was conducted on 75 school-aged children with a diagnosis of ADHD based on DSM-V criteria. Children were randomly allocated to receive either vitamin D3 (2000 IU/day) or a placebo for 3 months. Serum IL-6, TNF-*α,* and 25(OH) D were assessed before and after the intervention to determine the effects of vitamin D on the highlighted parameters.

**Results:**

Serum levels of 25(OH) D increased significantly in the vitamin D group (*P*=0.01). However, no significant differences in serum IL-6 and TNF-*α* were found between both groups at the baseline and at the end of the intervention.

**Conclusion:**

The findings revealed that vitamin D supplementation for 3 months is not efficacious in reducing inflammatory cytokines in children with ADHD. Further studies are required to confirm these results.

## 1. Introduction

Attention deficit hyperactivity disorder (ADHD) is one of the most prevalent psychiatric and developmental disorders among children and adolescents [1]. It features clinical impairments of hyperactivity, impulsivity, and inattention [2–6]. In addition, these children have challenges with school performance and independent socioeconomic factors [7, 8]. Psychiatric comorbidities of ADHD include aggression, mood disorders, and antisocial behavior [9, 10]. The overall prevalence of ADHD in school-aged children is estimated to be 3–5%, affecting 2–18% of children worldwide [11, 12], and it is estimated that three-quarters of these children retain ADHD symptoms in adulthood [1]. Despite considerable research, the underlying mechanisms leading to ADHD are poorly understood [12, 13]. Some studies indicated that abnormal immune functioning may play a substantial role in the etiology of this disorder. Meanwhile, ADHD has been suggested to contribute to an exaggerated central nervous system (CNS) and inflammatory response in a fetus as a result of maternal inflammation [14]. Furthermore, the elevated levels of proinflammatory cytokines such as interleukin IL-6 may be associated with the risk of ADHD.

Vitamin D has novel functions beyond its classical roles in bone metabolism. It might regulate brain functions [15] and has significant effects on neurodevelopmental diseases such as autism spectrum disorder, schizophrenia, and ADHD [16–19]. Several lines of evidence including the recent meta-analysis verify the lower serum vitamin D levels in children and adolescents with ADHD, compared to healthy controls [20–24]. Prior studies reported that vitamin D deficiency interferes with the brain development [25] andhas been associated with ADHD in children [20, 21, 23, 26, 27]. However, few studies have explored the association between vitamin D supplementation and symptoms of ADHD in children. On the other hand, vitamin D has been highlighted over the years due to its anti-inflammatory properties. In this direction, it can reduce the expression of inflammatory cytokines genes and plays regulatory roles in the immune system [28–30] and inflammation [31]. While methylphenidate is the first-line drug recommendation for ADHD to improve attitudes, other treatment procedures are of utmost importance [32]. Therefore, the aim of the present study was to assess the possible effects of vitamin D on TNF-*α* and IL-6 levels in children with ADHD.

## 2. Methods

### 2.1. Study Design

This randomized, double-blind, placebo-controlled clinical trial was conducted on 86 children aged 6–12, referred from Ziaeian Hospital (Tehran, Iran) between December 2015 and September 2016. The participants had different ethnicities including Persian, Tork, Mazani, Gilak, Lor, and Kord with yellowish skin tones. The study was approved by the Ethical Committee of Tehran University of Medical Sciences (IR.TUMS.REC.1394.1459) on 21 December, 2015 and was registered in the Iranian Registry ofClinical Trials under the registration number IRCT2016102324081N2. All of the participants' parents were informed about the study objective, and written informed consent was obtained.

### 2.2. Determination of Sample Size

Considering the design of prior studies based on the expected change of IL-6 and obtaining standard deviation, [33] sample size was estimated to achieve a power of 80% (1-*β* = 0.8), a type I error *α* = 0.05, and a 10% dropout rate. The total sample size of 80 (40 patients in each group) was calculated.

### 2.3. Study Participants

All 6–12-year-old patients admitted to this trial were screened by a psychiatrist for eligibility. Inclusion criteria were as follows: children with ADHD diagnosed less than one year according to the Diagnostic and Statistical Manual of Mental Disorders, Fifth Edition (DSM-V), methylphenidate consumption for less than one year, and body mass index (BMI) <95th percentile. Exclusion criteria were as follows: body mass index >95^th^ percentile; a history of diabetes, hypertension, or hyperthyroidism; infectious or respiratory diseases; digestive or cardiovascular diseases; liver or kidney diseases and allergy or neurological diseases; use of any medications or vitamin supplements within the 3 months before study enrollment.

### 2.4. Description of Intervention

Patients were randomly assigned to two groups to receive vitamin D3 or a placebo to investigate the therapeutic effects of vitamin D3 in children with ADHD. Random assignment was accomplished using a permuted-block randomization method; treatment and placebo group. Blood samples were taken at the beginning and the end of the intervention. Participants were considered compliant if they consumed more than 80% of the supplements. To follow patients' adherence, they were called once a week. Dietary information, including energy, macronutrients, and micronutrients, was obtained using a validated 24-hour food recall (for 3 days including 2 week days and one weekend day) filled in by a trained dietician at the beginning and the end of the study [34].

### 2.5. Assessment of Variables

At baseline, participants were interviewed to collect data regarding age, sex, birth weight, medications and supplements in use, and date of ADHD diagnosis. Anthropometric measurements, including weight and height, were carried out before and after the intervention. Weight was measured in a fasting state by the Seca scale to the nearest 0.1 kg with light clothing and no shoes, and height was recorded to the nearest 0.1 cm using a nonelastic meter fixed to a wall in a standing position. Birth weights were ascertained from their medical records. The sex-specific body mass index (BMI) percentiles were calculated using the SAS program for the 2000 CDC Growth Charts for the United States [35] and categorized as follows: underweight (BMI percentile <5), normal (BMI percentile 5–85), overweight (BMI percentile 85–95), and obese (BMI percentile >95). A validated questionnaire was used to quantify sunlight exposure. The total score was based on the duration and part of the body exposed to sunlight [36].

### 2.6. Randomization and Drug Allocation

Permuted-block randomization was used to divide patients randomly into two groups, so there was no difference according to age and gender. All participants and researchers, with the exception of the study statistician to generate randomization codes, were blinded to the intervention. Random allocation and rating of patients were conducted through a third person who was not involved in the study. 2000 IU of vitamin D has been recognized as an optimum dosage to prevent diseases without any adverse events [37, 38]. All participants, based on randomization, received two tablets of vitamin D3 (containing 1000 IU per tablet) or a placebo, every day for 3 months. Supplements were given to patients at the beginning and midpoint of the trial. The vitamin D3 and placebo tablets used in this study were purchased from Jalinous Company, Iran. The appearance of the vitamin D and placebo tablets was indistinguishable in terms of size, shape, color, and packaging. They were labeled as A or B and remained concealed in a sealed envelope until the end of the analysis. Patients were asked to take methylphenidate prescribed by their neurologist and not to take any other medications or supplements during the trial.

### 2.7. Laboratory Measurements

At the beginning and the end of the intervention, 10 cc of blood was drawn after a 12-hour overnight fast. Serum samples were separated and stored at -80°C. Serum 25-hydroxyvitamin D [25 (OH) D], IL-6, and TNF-*α* were assessed by applicable enzyme-linked immune sorbent assay (ELISA) kits (25 (OH) D: ELISA kit; DIA source: immuno assays S.A; the others: Bioassay Technology Laboratory, China) according to manufacturers' instructions.

### 2.8. Statistical Analysis

The analysis was performed using SPSS for Windows (SPSS, version 20; Chicago, IL, USA), and all data were presented as the mean ± SE (standard error). The normality of the parameter distribution was checked using the Kolmogorov–Smirnoff test. The independent sample *t*-test was used to compare the baseline characteristics and dietary intakes of the two groups. Covariance analysis (ANCOVA) adjusted for the baseline vitamin D was used to compare the means of variables between the two groups before and after the intervention. Comparison of qualitative variables was conducted by the chi-square test. *P* values ≤0.05 were considered statistically significant.

## 3. Results

### 3.1. Study Population and General Characteristics

Out of 86 enrollees, 5 patients in the vitamin D group were excluded from the study because of changing their physician (*n* = 2), no tendency to take methylphenidate (*n* = 2), and getting sick and stopping drug consumption (*n* = 1). In the placebo group, 6 patients were excluded due to their unwillingness to take methylphenidate. The study, then, was carried out with 75 patients. The baseline characteristics of the participants are presented in Table 1. There were no significant differences between the groups in terms of age, gender, weight, and birth weight (*P* > 0.05). Following the determination of obesity according to the BMI percentile, observations revealed no significant difference in BMI percentile between the two groups (*P*=0.98) (Table 2).

### 3.2. Dietary Intake

Dietary intakes of energy, carbohydrates, protein, fat, calcium, vitamin D, vitamin C, vitamin E, selenium, EPA, DHA, and beta-carotene did not differ significantly between the two groups (*P* > 0.05) (Table 3).

### 3.3. Circulating 25 (OH) D

Serum 25(OH) D increased significantly after the supplementation of vitamin D compared with the placebo at the end of the study (*P*=0.01) (Table 4).

### 3.4. Sun Exposure

The mean daily sunlight exposure scores of the vitamin D and placebo groups were 12.43 ± 1.38 and 13.58 ± 1.35, respectively, and no significant difference was detected between them (*P*=0.55).

### 3.5. Inflammatory Biomarkers

At the baseline, a non-significant difference was revealed in the levels of IL-6 and TNF-*α* between the placebo and vitamin D groups (*P*=0.46 and *P*=0.95, respectively) (Table 5). However, a significant difference between the treatment and placebo groups was observed at the vitamin D baseline (*P*=0.05). No significant differences in the serum of IL-6 and TNF-*α* were reported in both the groups at the end of the intervention when the analysis was adjusted for possible confounding factors as well (*P*=0.91 and *P*=0.76, respectively).

## 4. Discussion

In the current study, children with ADHD taking vitamin D supplementation for 3 months demonstrated a significant increase in serum levels of 25(OH)D. However, serum levels of IL-6 and TNF-*α* were not significantly influenced by vitamin D administration. Based on our knowledge, the present study is the first trial that investigates the effect of vitamin D supplementation on inflammatory cytokines in patients with ADHD.

There is a growing belief that vitamin D can decline the expression of inflammatory cytokines genes, [39] as well as decrease inflammatory markers through several putative mechanisms. Among all the pathways, recent studies have demonstrated that toll-like receptors (TLR) play an important role [40]. TLR4 activation triggers I*κ*B phosphorylation and degradation, resulting in the phosphorylation and translocation of NF-*κ*B [41, 42]. NF-*κ*B modulates the transcription of the extent of genes involved in inflammation, including TNF-*α* and IL-6 [43–46]. As a result, the main potential anti-inflammatory effects of vitamin D involve the promotion of negative feedback regulation of TLR4 and the inhibition of the NF-*κ*B pathway [40]. Another possible approach is the translocation of nuclear vitamin D receptors into mitochondria of certain cell types and the inhibition of calcium transition into this organelle. Consequently, the NF-*κ*B pathway will be controlled, [47] possibly causing a significant reduction in the expression of inflammatory biomarkers.

The relationship between vitamin D supplementation and inflammatory biomarkers has been examined in human and animal studies with contradictory results. In adults, based on a high dose (200,000 IU) of oral vitamin D to attenuate inflammation from sunburn, a significant decrease in serum level of TNF-*α* was indicated [48]. Similar to our findings, one assessment did not support the theory of association between vitamin D supplementation and serum TNF-*α* reduction [49]. Furthermore, intervention with vitamin D for equal or more than 12 weeks in a meta-analysis study by Calton et al. demonstrated that serum levels of IL-6 did not significantly decrease. This finding supports the hypothesis that older age predicts a significant reduction in IL-6, and a greater dosage of vitamin D may have the potential to improve inflammatory factors [50]. More so, in two animal studies evaluating the effect of vitamin D supplementation on IL-6 and TNF-*α* in rats, reduced levels of cytokines were also mentioned [51, 52].

Many studies have confirmed the relation between ADHD and inflammatory diseases and polymorphisms in inflammation-related genes, possibly due to the etiological role of inflammation in the development of ADHD [53]. In light of the ongoing debate about vitamin D status in patients with ADHD, several studies corroborated the lower serum vitamin D levels in them. The results of one clinical trial demonstrated lower levels of vitamin D in participants with ADHD [54]. Moreover, a vitamin D deficiency was reported [23] and then confirmed in a large sample (*n* = 1331) among patients with and without ADHD, aged 5–18 years [21]. The association between maternal 25(OH) D plasma levels in the 13th week of pregnancy and ADHD symptoms in children was shown in a cohort study [18]. However, in one experiment no association was found [55]. Considering the risk of ADHD observed with vitamin D deficiency, the aforementioned link between inflammatory markers with ADHD, the potential effect of vitamin D on these markers, and limiting and conflicting results, further studies are warranted. Notably, all participants were taking methylphenidate during the trial. It has been shown that a decline in dopamine activity occurs in children and adults with ADHD [56]. Thus, medical interventions such as methylphenidate were used to treat ADHD which acts by elevating the activity of the dopamine signaling pathway [56, 57]. On the other hand, two previously animal studies indicated that taking methylphenidate was positively correlated with increased inflammatory markers [58, 59]. Nevertheless, prompt future research without methylphenidate involvement appears to be effective in appraising the effect of vitamin D on inflammatory biomarkers.

Thus far, the scientific consensus has not been reached on the definition of the blood levels of 25(OH)D, indicating vitamin D deficiency, insufficiency, and sufficiency. For instance, despite adequate intake of vitamin D or sufficient exposure to sunlight, serum levels of 25(OH)D may be considered low, due to their conversion to the other forms of vitamin D such as 1,25(OH)D. It has been accepted that 25(OH)D at least 30 ng/ml or 75 nmol/L is relatively protective, whereas levels of 60 to 80 ng/ml would be enough to treat cancer, diabetes, and depression [60]. As we noted above, vitamin D supplementation in one systematic review did not induce a statistically significant decrease in inflammatory markers because serum 25(OH) D greater than 80 nmol/l may be considered a target level to decrease inflammatory cytokines [50]. Given that in the current study, the serum levels of 25 (OH) D were elevated to 33.44 nmol/l.m, and anti-inflammatory effects of vitamin D are possibly obtained in serum levels over 50 nmol/L, as well as larger trials with elevating supplementation are suggested. Since vitamin E in combination with vitamin C as an antioxidant may attenuate elevated plasma IL-6 levels, [61] vitamin D supplementation with an antioxidant such as vitamin C might possibly have more impact.

### 4.1. Limitations

The short duration of supplementation and using methylphenidate along with vitamin D are two of the main limitations of this study. Although taking methylphenidate positively influences the compliance of participants, it may increase the TNF-*α* levels [62]. On the other hand, vitamin D supplementation for 3 months did not elevate serum 25(OH) D to optimum levels. Therefore, further investigation of long-term vitamin D intervention without methylphenidate is suggested. More so, several experiments exhibit that the serum level of vitamin D is not regarded as an accurate measure of vitamin D status. PTH measurement, thus, is proposed to assess its suppression [63]. As an additional limitation, we did not consider seasonality and UV exposure as well as oral health status. Evidence shows that children with ADHD might have poor oral health status such as dental caries and periodontal disease which can elevate the inflammatory biomarkers. Subsequently, evaluation of oral health status may provide more accurate data in this group of patients [64–66].

## 5. Conclusions

Our study failed to find a favorable effect of 3 months of supplementation with vitamin D on inflammatory cytokines (IL-6 and TNF-*α*) in school-aged children with ADHD. Conducting further studies with different doses of vitamin D and various designs in addition to differences in the characteristics of participants is highly recommended.

## Figures and Tables

**Table 1:** Baseline characteristics between treatment and placebo groups.

Variable	Vitamin D (*n* = 37)	Placebo-control (*n* = 38)	*P* value
^*∗*^Gender *n* (%)			
Male	28 (75.7)	24 (63.2)	
Female	9 (24.3)	14 (36.8)	0.24
Age (month)	100.65 (3.55)	106.18 (4.03)	0.30
Actual height (cm)	135.81(2.26)	137.10 (1.81)	0.65
Actual weight (kg)	32.15 (2.13)	32.60 (1.69)	0.86
Birth weight (gr)	3237.30 (92.76)	3115.79 (110.34)	0.38
Sun exposure (daily score)	12.43 (1.38)	13.58 (1.35)	0.55

Independent samples *t*-test; mean ± SD; SD: standard error.  ^*∗*^ Chi-square; number (percent).

**Table 2:** Comparison of the BMI between treatment and placebo groups at the baseline.

Variable		Vitamin D	Placebo-control	*P* value
BMI (kg/m^2^)	<5	8 (21.6)	8 (21.1)	0.98
	5–85	19 (51.4)	19 (50.0)	
	85–95	10 (27.0)	11 (28.9)	
	95<	0	0	

chi-square test; number (percent).

**Table 3:** Comparison of dietary intake between treatment and placebo groups at the baseline.

Variable per day (%)	Vitamin D	Placebo-control	*P* value
Energy (Kcal)	1835.13 (61.35)	1822.84 (32.83)	0.85
CHO (g)	276.10 (36.01)	231.42 (6.35)	0.22
PRO (g)	58.77 (2.699)	55.11 (2.14)	0.29
FAT (g)	78.57 (2.79)	78.32 (2.31)	0.94
Vitamin E (mg/L)	23.82 (1.22)	23.05 (0.96)	0.62
Vitamin D (*μ*g)	2.95 (1.85)	2.69 (2.14)	0.92
Vitamin C (mg)	67.29 (7.78)	74.42 (12.02)	0.62
Selenium (*μ*g)	0.07 (0.03)	0.03 (0.004)	0.30
B-carotene (*μ*g)	798.46 (131.13)	697.30 (113.44)	0.56
EPA (g)	0.01 (0.005)	0.01 (0.005)	0.96
DHA (g)	0.57 (0.54)	0.03 (0.01)	0.31
Calcium (mg)	510.89 (69.16)	552.66 (34.76)	0.58

^*∗*^Independent samples *t*-test; mean ± SD; SD: standard error.

**Table 4:** Comparison of the mean difference of variables at the baseline and end of the study between the two groups.

	Placebo-control		Vitamin D		*P* value
	Baseline	After 3 month	Baseline	After 3 month	3
IL-6 (pg/ml)	107.63 (11.96)	119.24 (18.12)	94.92 (12.44)	101.94 (16.61)	0.913
TNF- *α* (pg/ml)	116.22 (18.39)	170.84 (29.85)	114.58 (25.15)	163.86 (31.74)	0.766
Vitamin					
D (ng/ml)	15.99 (1.88)	15.99 (1.88)	23.52 (1.75)	33.44 (2.14)	0.01

Univariate covariance analysis (ANCOVA) adjusted for the baseline vitamin D; ^*∗∗*^mean ± standard error; IL-6: interleukin-6; TNF- *α*: tumor necrosis factor alpha.

**Table 5:** Comparison of the baseline variable between treatment and placebo groups.

Variable	Placebo-control	Vitamin D	*P* value
IL-6 (pg/ml)	107.63 (11.96)	94.92 (12.44)	0.46
TNF- *α* (pg/ml)	116.22 (18.39)	114.58 (25.15)	0.95
Vitamin D (ng/ml)	15.99 (1.88)	23.52 (1.75)	0.05

Independent *t*-test; ^*∗∗*^ mean ± standard error; IL-6: interleukin-6; TNF- *α*: tumor necrosis factor alpha.

## Data Availability

The data used to support the findings of this study are available from the corresponding author upon reasonable request.

## Abbreviations

ADHD: (Attention-deficit/hyperactivity disorder)
IL-6: (Interleukin-6)
TNF-*α*: (Tumor necrosis factor-alpha)
DSM-V: (Diagnostic and statistical manual of mental disorders 5th)
NF-*κ*B: (Nuclear factor kappa B)
